# *Strongyloides stercoralis*: A Plea for Action

**DOI:** 10.1371/journal.pntd.0002214

**Published:** 2013-05-09

**Authors:** Zeno Bisoffi, Dora Buonfrate, Antonio Montresor, Ana Requena-Méndez, Jose Muñoz, Alejandro J. Krolewiecki, Eduardo Gotuzzo, Maria Alejandra Mena, Peter L. Chiodini, Mariella Anselmi, Juan Moreira, Marco Albonico

**Affiliations:** 1 Centre for Tropical Diseases (CTD), Sacro Cuore–Don Calabria Hospital, Negrar, Verona, Italy; 2 Coordinating resources to assess and improve health status of migrants from Latin America (COHEMI) project study group, European Commission, Health Cooperation Work Programme, FP7 (GA-261495); 3 Department of Control of Neglected Tropical Diseases, World Health Organization, Geneva, Switzerland; 4 Barcelona Centre for International Health Research (CRESIB, Hospital Clínic-Universitat de Barcelona), Barcelona, Spain; 5 Instituto de Investigaciones en Enfermedades Tropicales, Universidad Nacional de Salta, Salta, Argentina; 6 Instituto de Medicina Tropical Alexander von Humboldt, Universidad Peruana Cayetano Heredia, Lima, Peru; 7 Hospital for Tropical Diseases and London School of Hygiene & Tropical Medicine, London, United Kingdom; 8 Centre for Community Epidemiology and Tropical Medicine (CECOMET), Esmeraldas, Ecuador; 9 Ivo de Carneri Foundation, Milano, Italy; Centers for Disease Control and Prevention, United States of America

Strongyloidiasis remains an underestimated public health problem, just as it was at the dawn of last century.

In 1901, Professor William Sydney Thayer published a review, “On the Occurrence of *Strongyloides intestinalis* in the United States,” concluding: “…one may be justified in emphasizing the following points:

1. “Diarrhoea associated with the presence of *Strongyloides intestinalis* occurs in the United States.
2. “The observation, in the Johns Hopkins Hospital, of three cases within three years, […] suggests that this parasite may be more frequent than hitherto been supposed.
3. […]
4. […]
5. “More systematic examinations of the faeces both in public clinics and in private practice are much to be desired.” [1]

More than one century later, the key issues regarding this parasite (subsequently renamed *Strongyloides stercoralis*) are essentially the same, and although researchers have recently given more attention to this infection, systematic action plans still lag behind. There is widespread agreement in the scientific community that its prevalence is largely underestimated [2]. The current estimate of 30 to 100 million infected persons in the world dates back to review articles published between 1989 and 1996 [3], [4], and is cited by most subsequent papers. These figures were mostly based on surveys aimed at defining the prevalence of parasitic infections, without using adequate diagnostic techniques for *S. stercoralis*. For example, Kato-Katz, a technique that is commonly used in surveys aiming to assess intestinal helminth infections [5], is poorly sensitive for this parasite. Larvae of *S. stercoralis* in stool are often scanty, and therefore they are most often missed by this technique that examines a small amount of faeces (between 20 and 50 mg, depending on the template). Larvae can be detected by this technique only occasionally, when the larval output is particularly high [6]. More reliable prevalence estimates have been made by geographically confined surveys, using alternative faecal-based diagnostic methods that are much more sensitive such as Baermann or Koga agar plate culture [7], [8]. Serology (ELISA or IFAT) is even more sensitive, but its specificity is less well defined. Problems of cross-reactivity seem to arise especially in areas where other nematodes, particularly filariae, are also endemic. New and promising tools such as serologic methods based on recombinant antigens or PCR are also available in some referral centers. However, the optimal diagnostic strategy, both for epidemiological surveys and for individual diagnosis and screening, has yet to be defined and certainly deserves further research [9].

Nevertheless, global prevalence estimates should probably be revised, based on the studies using diagnostic techniques that are better suited to *S. stercoralis*.

If we take hookworm as a comparison, as they have the same route of infection as *S. stercoralis*, we observe that surveys using (for *S. stercoralis*) Baermann and/or coproculture report a ratio of *S. stercoralis* to hookworm of 1/4 to 1/1 or more [7], [10]–[14]. In a recent study comparing the Kato-Katz method and the spontaneous sedimentation in tube technique (SSTT) for the diagnosis of intestinal parasites in the Amazonian basin of Peru [7], the researchers found the same prevalence with both techniques for hookworm (14%) as well as for *Ascaris lumbricoides* and *Trichuris trichiura* (both 5%), while for *S. stercoralis* the prevalence was 0% with Kato-Katz, versus 16% with the alternative method, and 22% if agar plate culture was added. The ratio of *S. stercoralis* to other helminthes would be even higher if we had to consider serologic surveys [12], [15], [16], and the figures would be different if multiple sampling were to be used [8]. If we refer to the current estimate of 740 million people infected with hookworm globally [17], a prevalence of at least 370 million people infected with *S. stercoralis* worldwide seems a more reasonable (and probably still conservative) figure. Better tools are needed for a more correct estimation of *S. stercoralis* prevalence, using at least one of the best available diagnostic methods in stools such as Baermann or Koga agar plate culture, and adding when possible an accurate serologic test.

Furthermore, the burden of mortality and morbidity associated with this parasite is poorly defined. Going back to 1933, we read: “history of the case supports the considerable accumulated evidence that strongyloidiasis may give rise to severe symptoms” [18].

**Figure 1:**
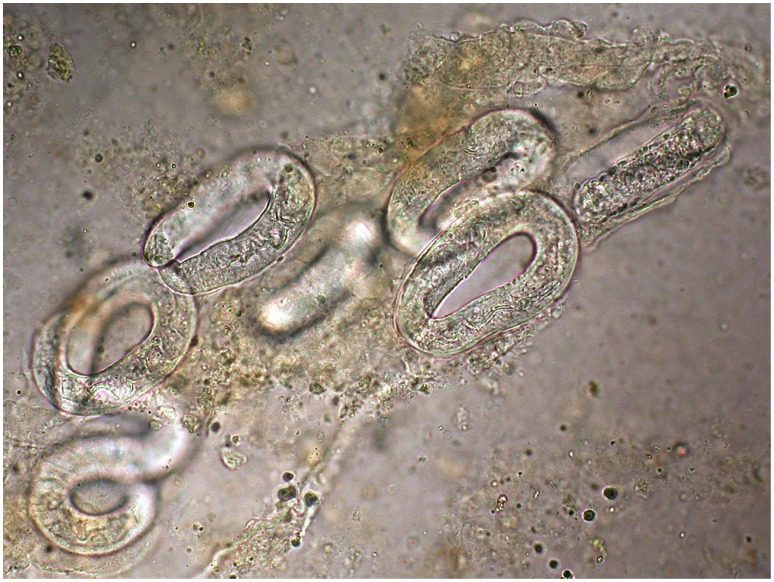
Embryonated eggs of *Strongyloides stercoralis* in bronchial fluid from a fatal case of disseminated strongyloidiasis (photo by Maria Gobbo, CTD Negrar, Verona).

We now know that disseminated strongyloidiasis (Figure 1) is a life-threatening condition for immunosuppressed patients, with death often occurring in a few days. In patients with hematologic malignancies (especially lymphoma), medically induced immune suppression (e.g., transplant recipients), or under corticosteroids (sometimes even for the symptoms caused by an unrecognized infection with *S. stercoralis*), the autoinfection cycle of this parasite becomes overwhelming, with larvae invading virtually all organs and tissues [19]. Nevertheless, the magnitude of this risk is unclear. How many chronically infected people receiving steroids, or exposed for any reason to one of the other well-known risk factors for severe disease, are likely to develop dissemination? Most cases have been reported in western countries or in other affluent countries where the prevalence of the infection is low and about half the cases are seen in migrants [20]. Even there, severe cases often remain undiagnosed, as the clinical presentation is variable and nonspecific. In most of African, Asian, and Latin American countries, reports of severe and fatal strongyloidiasis are lacking or exceedingly rare, meaning that most cases must be missed. Moreover, in some of them HTLV-1 infection, one of the recognized risk factors for disseminated disease, is relatively common [21]. Furthermore, in countries in economic transition, given the increase in chronic medical conditions and malignancies, as well as in the availability of potentially dangerous treatments, the number of immunosuppressed patients exposed to the risk of severe or fatal complications of an unrecognized, chronic *S. stercoralis* infection is likely to grow at a fast rate. More generally, the impact of this parasite on the total morbidity and mortality in the low-middle income countries is poorly known. In a recent study in Côte d'Ivoire comparing self-reported morbidity of *S. stercoralis* versus hookworm, a trend toward worse health conditions, particularly in those infected with *S. stercoralis*, was observed. Moreover, the perceived health impact of strongyloidiasis was greater than that of hookworm, mainly concerning abdominal and respiratory symptoms and skin problems [14].

A further concern is that, while the prevalence of the other soil-transmitted helminths (STH) is declining, mass treatment campaigns are unlikely to impact on *S. stercoralis*, except in countries where ivermectin, the drug of choice for strongyloidiasis, has been introduced for control/elimination of onchocerciasis and lymphatic filariasis [22], [23].

Therapy remains an area of partial uncertainty, too, as the optimal dosage schedule of ivermectin has yet to be defined [24], [25]. Solely reducing the worm burden, which may be an acceptable goal for other helminths, is not enough in this case, as this worm is capable of replicating itself in the host due to its peculiar autoinfection cycle. Moreover and for the same reason, this infection can last lifelong even in the absence of a reinfection, if not adequately treated [19]. A number of randomized clinical trials have been carried out, showing that ivermectin is the drug of choice, and a single dose is highly effective (over 90% in most studies) [26], [27]. However, drug efficacy may have been overestimated, as faecal-based methods alone have been used to assess cure in almost all studies. Multiple doses may be necessary to obtain the goal of eradication in a patient, and the current indications by WHO refer to a schedule of two consecutive days as a possible alternative to the single dose [28]. However, a single dose may be appropriate for mass treatment, as even the clinical trial with the most strict criteria of cure (combining serology with feacal-based diagnostic methods) documented eradication of the infection in almost 70% of patients [24]. Currently, ivermectin is being used for mass treatment of filariasis: estimating the impact on strongyloidiasis in countries where this drug has been used in mass treatment would be useful for planning control actions. A recent study in Zanzibar showed a dramatic impact on STH prevalence following mass treatment with ivermectin plus albendazole for control of lymphatic filariasis; moreover, a tremendous reduction was observed in the incidence of scabies, too, which is known to respond well to ivermectin [29]. Further studies comparing areas where NTD control strategies include filariasis with areas where ivermectin has not been used, together with a more reliable mapping of *S. stercoralis* prevalence, would be crucial to guide focused actions for the control of this parasite.

In summary, many gaps in knowledge remain and should be addressed by future research. Networking is crucial among the relatively few researchers interested in this neglected infection to improve coordination and optimize resources. An information sharing point has been recently opened on the WHO website for this purpose. Meanwhile, we already know a great deal. We know that this parasite kills; we know that the infection can last lifelong in the absence of effective treatment; we know that the prevalence is probably much higher than previously estimated; we know that current regimens for mass treatment of STH are not adequate unless they include ivermectin; we know that the latter is the drug of choice for strongyloidiasis; and we have substantial experience in the large-scale use of this drug.

Despite many remaining grey areas, the existing evidence calls for the following urgent, essential steps to be taken:

Prevalence studies of STH should also target *S. stercoralis* using adequate diagnostic tools, and include comparative studies in areas where ivermectin has long been used for onchocerciasis and lymphatic filariasis control/elimination. This should ideally include development of new diagnostics for use in field settings.
Donors and funding agencies interested in supporting NTD research and control should not ignore *S. stercoralis*, currently a Cinderella in this arena.
A forum of experts should establish prevalence thresholds with the currently available diagnostic tools in order to define *S. stercoralis* as a public health problem, and propose control strategies including mass treatment regimens.
Ivermectin should be made available for mass treatment in countries/areas with high prevalence.
Adequate screening and treatment strategies should be the rule for patients at risk of immunosuppression, both in low-middle and high income countries. More sensitive tests for parasitological cure are required.

We know enough to call for action now.
